# Effect of levothyroxine replacement therapy in patients with subclinical hypothyroidism and chronic heart failure: A systematic review

**DOI:** 10.3389/fendo.2022.1013641

**Published:** 2022-11-15

**Authors:** Vincenzo Triggiani, Antonio Cittadini, Giuseppe Lisco

**Affiliations:** ^1^ Interdisciplinary Department of Internal Medicine, University of Bari “A. Moro”, Bari, Italy; ^2^ Department of Translational Medical Sciences, Federico II University, Naples, Italy

**Keywords:** subclinical hypothyroidism, chronic heart failure, levothyroxine, all-cause mortality, cardiovascular mortality

## Abstract

**Background:**

Chronic heart failure (CHF) affects the health care system with high social and economic costs due to recurrent hospital admissions or frequent ambulatory reassessments. Subclinical hypothyroidism (SCH) is commonly observed in patients with CHF and negatively affects myocardial function and remodeling and, ultimately, increases the risk of hospitalizations and all-cause and cardiovascular (CV) mortality. The role of levothyroxine replacement on relevant CV outcomes in patients with SCH and CHF is unclear.

**Objective:**

To assess the effect of levothyroxine (compared to placebo or no treatment) on the incidence of all-cause and CV mortality, major adverse CV events, and heart failure in patients with SCH and CHF.

**Methods:**

PubMed/MEDLINE, Cochrane Library, and ClinicalTrial.gov were searched for randomized clinical trials, non-randomized observational, multicentric, and comparative studies. No language restrictions were included. After duplicate removal, articles were screened and extracted for the synthesis according to a hierarchical strategy that included title, abstract, and full-text appraisal. The risk of bias was assessed by RoB2 and ROBIN-I tools. The Grading of Recommendations Assessment, Development and Evaluation (GRADE) methodology was applied to rate the quality of evidence and grade the strength of recommendations.

**Results:**

Two trials were included in the systematic review with considerable indirectness and inaccuracy that down-graded the level of evidence.

**Discussion:**

No evidence supports the use of levothyroxine for treating SCH in CHF due to the lack of reliable and well-designed clinical trials.

**Conclusion:**

CV outcome and dose-response trials are needed to understand better the role of levothyroxine replacement treatment for a safer prescription in this clinical setting.

## Background

Chronic heart failure (CHF) is characterized by structural and functional cardiac abnormalities, confirmed by high circulating levels of natriuretic peptide or signs and related symptoms of either pulmonary or systemic congestion (1). Endocrine disorders are frequently observed in CHF, including thyroid dysfunction (2–4). Current guidelines suggest to check thyroid hormones in patients with CHF to prevent possible adverse events (5, 6), thus raising the importance of prompt recognition and management of these disorders. However, the efficacy and safety of medical management of thyroid disorders in CHF are poorly investigated, and pharmacological treatments are based on general recommendations. Among thyroid disorders, hypothyroidism is a frequent comorbidity in CHF with non-negligible detrimental CV consequences (7, 8).

Herein we provide an overview of the current status of knowledge on the diagnosis and clinical significance of subclinical hypothyroidism (SCH) and a systematic review of the effect of levothyroxine treatment on cardiovascular outcomes in patients with SCH and CHF.

## Hypothyroidism

SCH is defined as an elevated serum thyrotropin hormone (TSH) level with values of free thyroxine (fT4) and free triiodothyronine (fT3) within the normal range of reference (9). Primary overt hypothyroidism (OH) is characterized by a TSH value above the higher limit of the normal range with fT4 levels below the lower limit of the normal range. TSH elevation predicts the future development of hypothyroidism, especially when thyroid antibodies are detected (10).

The prevalence of SCH and OH increases with aging (11) along with the prevalence of CHF (12). Therefore, SCH and OH are frequently observed in patients with CHF (13). In some cases, chronic assumption of amiodarone, a class III antiarrhythmic medication to prevent ventricular arrhythmias, may be another cause of hypothyroidism in CHF (14). Thyroid hormones, mostly T3, are essential to regulating the expression of genes involved in myocardial contractility and pump efficiency (15). Chronic deficiency of thyroid hormones contributes to myocardial fibrosis and predisposes to coronary blood flow failure (16, 17). Moreover, hypothyroidism prompts endothelial dysfunction, atherosclerosis, systemic inflammation, hypercholesterolemia, and insulin resistance, which are well-known risk factors for CHF progression (18, 19). Observational studies confirmed a direct relation between TSH value and poor outcomes in patients with CHF (i.e., hospital admission due to acute decompensation, all-cause mortality, and cardiovascular mortality) (7, 20, 21).

Given this evidence, levothyroxine may improve cardiovascular outcomes in patients with CHF and hypothyroidism, but it remains unclear when to start replacement and what is the optimal TSH target. In one observational study, the mortality rate was higher in the lowest (<0.1 mUI/L) and highest TSH categories (>4 mUI/L) than in the normal range of TSH (0.4 – 4 mUI/L). In addition, the risk of heart failure was elevated when TSH was below 0.4 mUI/L and above 10 mUI/L, especially in women and older patients. No increase in the risk of cardiovascular events, atrial fibrillation, and fracture across TSH concentrations was observed (22). In a 12-month prospective study, echocardiographic parameters, biomarkers as well as the risk of worsening heart failure or death deteriorated in patients who exhibited worsening uncontrolled hypothyroidism compared to those with stable normal o moderately elevated TSH (23). No improvement in cardiovascular outcomes was found in patients with SCH, especially in the elderly (24, 25).

## Subclinical thyroid dysfunctions in CHF

A large amount of evidence suggests that hypothyroidism is linked to cardiovascular diseases (CVDs). The underlying mechanisms explaining this association are related to impaired lipid metabolism, low-grade inflammation, endothelial dysfunction, hyperhomocysteinemia, and hypercoagulative state (26–28). This association is relevant in younger patients with OH, hence suggesting that the overall exposure and severity of hypothyroidism drive detrimental CV consequences (29).

SCH is accompanied by similar CV hallmarks (30), and it is associated with an increased risk of CHF among older adults exhibiting TSH values >7 mIU/L (31, 32). SCH than euthyroidism is associated with impaired maximum oxygen consumption during physical exercise and increased pulmonary pressure leading to intolerance to physical exercise (33).

Given the importance of thyroid hormones in regulating the mechanisms that maintain cardiovascular health, such as lipids, endothelial function, blood pressure homeostasis, and myocardial remodeling, both hypo- and hyperthyroidism may be implicated in the pathogenesis of cardiovascular adverse events (34, 35).

## Levothyroxine supplementation in patients with subclinical hypothyroidism and CHF

Although epidemiological data suggest that subclinical hypothyroidism may negatively affect cardiovascular outcomes in patients with CHF, the role of levothyroxine replacement in improving these outcomes or not should be better elucidated (36).

More precisely, we conceived this review in order to respond to the following questions:

- *Does levothyroxine replacement therapy compared to placebo or no treatment improve cardiovascular outcomes in patients with CHF and subclinical hypothyroidism?* (**Table 1**).

**Table 1:** Does levothyroxine replacement improve cardiovascular outcomes in patients with CHF? **PICO**.

**Population**	Patients with established heart failure and primary subclinical hypothyroidism
**Intervention**	Levothyroxine replacement
**Comparator**	Placebo or no treatment
**Outcomes**	Cardiovascular and all-cause mortality; cardiovascular events (both fatal and non-fatal); heart failure

The study population included adult patients (age >18 years) with an established diagnosis of CHF. Subclinical hypothyroidism was defined as 4.5 < TSH < 20 mUI/mL with normal values of fT4 and fT3.

### Methods

To this aim, a systematic search in PubMed/MEDLINE, Cochrane Library, and ClinicalTrials.gov were carried out by using the following strategies:

- *“(levothyroxine) AND ((heart failure) OR (chronic heart failure))”.*

**Figure 1:**
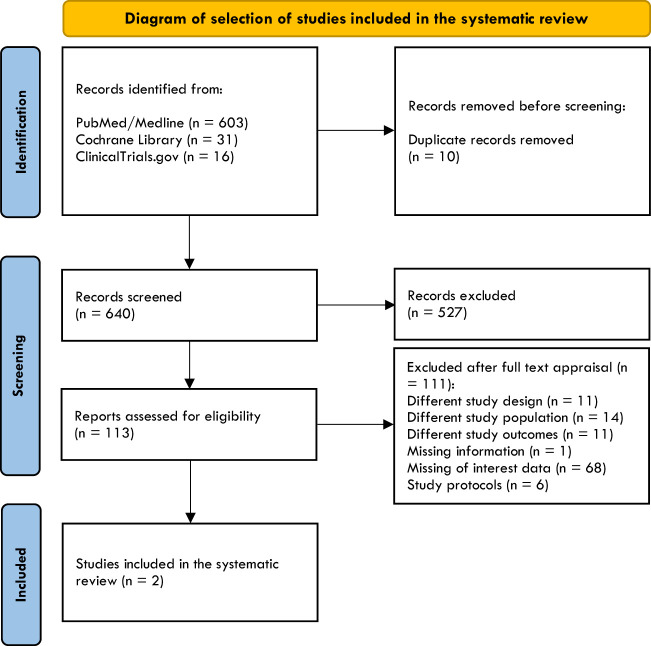
Diagram of selection of included studies.

Six-hundred-three results were found after searching PubMed/MEDLINE, 31 were found in the Cochrane Library, and 16 in ClinicalTrials.gov. The search strategy was restricted to randomized clinical trials (RTCs), non-randomized observational, multicentric, and comparative studies. No language restrictions were included. After duplicate removal, articles were screened and extracted for the synthesis according to a hierarchical strategy that included title, abstract, and full-text appraisal. The flow diagram summarizing the process of identification of studies has shown in **Figure 1**.

As pre-specified, the risk of bias in included studies was assessed by RoB2 for individual randomized, parallel-group trials (37), and ROBINS I for non-randomized observational cohort studies (38). The Grading of Recommendations Assessment, Development and Evaluation (GRADE) approach was applied for rating the quality of evidence and possibly grading the strength of recommendations.

### Results

Data from excluded studies are listed in more detail in supplemental materials. Only two studies were finally included in the systematic review (**Table 2**). Zijlstra et al. carried out a RCT deriving from a pre-specified combined analysis of two existing study protocols (39). The median age of study participants was 75 years, and the trial included a total of 842 patients, of whom 732 from the TRUST (age >64) and 112 from the IEMO80 plus trial (age >80). Subclinical hypothyroidism was as TSH value ranging from 4.6 to 19.9 mUI/L and confirmed in two different measurements from three months to three years apart. Over one-third of participants had a background established CVD, including CHF. Participants were randomized to receive levothyroxine at starting dose of 25 or 50 μg per day or placebo (1:1). A 1-year follow-up was carried out with more than 85% of participants completing the study. In Einfeldt et al., data were collected from five national (Danish) registries to carry out a retrospective cohort study (40). The diagnosis of heart failure was reported for each patient after the first-time heart failure-related hospitalization through the period 1997 – 2012. The registries also contain information about all diagnosed diseases and prescribed medications. Patients with hypothyroidism were considered both those assuming levothyroxine at baseline and those in which levothyroxine was prescribed during the follow-up. The mean age of patients was 70.7 years, and around one-third of them had a cardiovascular background disease (31%).

**Table 2:** Summary of included studies’ details.

Study(year)	Type of study	Definition of CHF	Definition of SCH	End-points	Participants and treatments	Median age (years)	Comorbidities	Established CVD	Follow-up (years)	Completion rate	Starting dose (µg)	Baseline TSH (mUI/L)	Final TSH (mUI/L)
Zijlstra et al. (2021)	Pre-specified combined analysis (TRUST, IEMO80+) RCT	Missing	4.6 < TSH^*^ < 19.9 mUI/L	All-cause and CV mortality Fatal and non-fatal CV events CV adverse events^**^	842 (53.3% women) Placebo 422 Levothyroxine 420 Randomization (1:1)	75.0 56.9% >80	Ischemic heart disease Stroke or transient ischemic attack Heart failure Peripheral vascular disease Revascularization or atrial fibrillation.	302 (35.9%)	1	Placebo 368 (87.2%) Levothyroxine 364 (86.4%)	50 (83.8%) 25 (16.2%)	6.38 ± 5.7	Placebo 5.66 ± 3.3 Levothyroxine 3.66 ± 2.1
Einfeldt et al. (2019)	Retrospective cohort study (Danish Registries)	Diagnosis of heart failure (ICD-10: I50)^***^	Missing	All-cause mortality Fatal and non-fatal CV events Cardiovascular death, MACE (CV death, fatal or non-fatal infarction, or stroke)	224,670 (53% men) Levothyroxine at Baseline (6,560) Levothyroxine During Follow-Up (9,007) No treatment (209,103)	70.7	Ischemic heart disease Atrial fibrillation Diabetes, Stroke	68,929 (31%)	4.8	n.a.	Missing	Missing	Missing

n.a., not applicable.

^*^ TSH confirmed in two determinations between 3 months and 3 years apart.

^**^ CV events included: myocardial infarction, stroke, amputations due to peripheral vascular disease, revascularizations for atherosclerotic vascular disease (including acute coronary syndrome), and heart failure hospitalizations. CV adverse events included: new-onset atrial fibrillation and new-onset heart failure.

Secondary outcomes included blood pressure, heart rate, and body weight, which were measured as positive signals of TSH change. *** Adults who received the diagnosis of heart failure with a first-time heart failure-related hospitalization in the period 1997 – 2012. The diagnosis of HF (ICD-10: I50) was validated with a specificity of 99%, suggesting that the diagnosis from the Danish National Registry is sufficiently high also to collect and analyze data for scientific purposes.

In Zijlstra et al., no statistically relevant differences in terms of either CV benefits or harms were found among patients assuming levothyroxine versus placebo. A total of 44 fatal and non-fatal CV events were observed after a median follow-up of 17 months without any relevant difference between the two groups. Twenty-one deaths for any cause occurred during the follow-up (of which four cardiovascular deaths) without any relevant differences between the two groups. Similarly, the cases of new-onset atrial fibrillation and heart failure were equally distributed between the two groups (**Table 3**). Subgroup analyses showed that these risks were similar also irrespective to baseline CV history and age of participants. After data extraction from Einfeldt et al. and analyses (calculation of the relative risk for each event, IC 95%, and p-value <.05 as statistical significance), the study event rates were lower among patients who were assuming levothyroxine (cumulative of those assuming levothyroxine at baseline and those who started levothyroxine assumption during the follow-up) compared to patients not treated with levothyroxine (**Table 4**). Despite the statistical relevance of the results, probably attributable to the large sample dimension, the clinical importance of these effects could be limited, especially for all-cause mortality, cardiovascular death, and myocardial infarction. A more relevant result could be attributable to the possible reduction in the number of observed major adverse cardiovascular events (MACE). However, in the entire cohort, a total of 147,253 patients died, and the authors calculated an increased risk of incidence of all-cause mortality (IRR 1.25; 95% CI, 1.21 to 1.29; IRR 1.13; 95% CI, 1.10 to 1.16), cardiovascular death (IRR 1.23; 95% CI, 1.18 to 1.27; IRR 1.11; 95% CI, 1.08 to 1.15), and a composite of cardiovascular death, fatal or non-fatal MI, or stroke or MACE (IRR 1.26; 95% CI, 1.22 to 1.31; IRR 1.05; 95% CI, 1.02 to 1.09) for treatment ongoing at baseline and initiated during follow-up, respectively. Moreover, an increased risk of incidence of myocardial infarction (IRR 1.32; 95% CI, 1.23 to 1.41) was observed for ongoing treatment with levothyroxine at baseline, while reduced risk (IRR 0.87; 95% CI, 0.81 to 0.93) was observed for incident treatment.

**Table 3:** Levothyroxine versus placebo for improving CV outcomes in patients with SCH and CHF (Zijlstra LE, et al., 2021).

Certainty assessment									Summary of findings		
Participants (studies) Follow-up	Risk of bias	Inconsistency	Indirectness	Imprecision	Publication bias	Overall certainty of evidence	Study event rates (%)		Relative effect (95% CI)	Anticipated absolute effects	
							With Placebo	With Levothyroxine		Risk with Placebo	Risk difference with Levothyroxine
**All-cause mortality**											
843 (1 RCT)	not serious	not serious	serious^a^	very serious^b,c,d^	none	⊕○○○ Very low	9/429 (2.1%)	12/414 (2.9%)	**RR 1.38** (0.59 to 3.24)	21 per 1.000	**8 more per 1.000** (from 9 fewer to 47 more)
**Fatal and non-fatal CV events**											
846 (1 RCT)	not serious	not serious	serious^a^	very serious^b,c,d^	none	⊕○○○ Very low	25/424 (5.9%)	19/422 (4.5%)	**RR 0.76** (0.43 to 1.37)	59 per 1.000	**14 fewer per 1.000** (from 34 fewer to 22 more)
**Serious adverse events**											
843 (1 RCT)	not serious	not serious	serious^a^	very serious^b,c,d,e^	none	⊕○○○ Very low	116/422 (27.5%)	90/421 (21.4%)	**RR 0.78** (0.61 to 0.99)	275 per 1.000	**60 fewer per 1.000** (from 107 fewer to 3 fewer)
**New onset atrial fibrillation**											
840 (1 RCT)	not serious	not serious	Serious^a^	very serious^b,c,d,e^	none	⊕○○○ Very low	15/417 (3.6%)	11/423 (2.6%)	**RR 0.72** (0.34 to 1.56)	36 per 1.000	**10 fewer per 1.000** (from 24 fewer to 20 more)
**New onset heart failure**											
829 (1 RCT)	not serious	not serious	serious^a^	very serious^b,c,d,e^	none	⊕○○○ Very low	9/429 (2.1%)	4/400 (1.0%)	**RR 0.48** (0.15 to 1.54)	21 per 1.000	**11 fewer per 1.000** (from 18 fewer to 11 more)

CI: confidence interval: RR: risk ratio

Explanations

a. Background prevalence of heart failure is low.

b. Sample size is underpowered.

c. The number of CV events is very low.

d. Short follow-up (considering the study objective).

e. Confidence interval includes potential for relevant benefit or harm.

The bolded text in this table emphasizes the results.

**Table 4:** Levothyroxine versus no treatment for improving CV outcomes in patients with SCH and CHF (Einfeldt MN, et al., 2019).

Certainty assessment							Summary of findings				
Participants (studies) Follow-up	Risk of bias	Inconsistency	Indirectness	Imprecision	Publication bias	Overall certainty of evidence	Study event rates (%)		Relative effect (95% CI)	Anticipated absolute effects	
							With [No treatment]	With [Levothyroxine]		Risk with [No treatment]	Risk difference with [Levothyroxine]
**All-cause mortality**											
224670 (1 observational study)	not serious	not serious	very serious^a,b,c^	serious^d^	none	⊕○○○ Very low	138103/209103 (66.0%)	9150/15567 (58.8%)	**RR 0.89** (0.88 to 0.90)	660 per 1.000	**73 fewer per 1.000** (from 79 fewer toM 66 fewer)
**CV death**											
224670 (1 observational study)	not serious	not serious	very serious^a,b,c^	serious^d^	none	⊕○○○ Very low	96575/209103 (46.2%)	6243/15567 (40.1%)	**RR 0.87** (0.85 to 0.89)	462 per 1.000	**60 fewer per 1.000** (from 69 fewer to 51 fewer)
**Myocardial infarction**											
224670 (1 observational study)	not serious	not serious	very serious^a, b, c^	serious^d^	none	⊕○○○ Very low	34023/209103 (16.3%)	1702/15567 (10.9%)	**RR 0.67** (0.64 to 0.70)	163 per 1.000	**54 fewer per 1.000** (from 59 fewer to 49 fewer)
**MACE**											
224670 (1 observational study)	not serious	not serious	very serious^a,b,c^	not serious	none	⊕○○○ Low	116010/209103 (55.5%)	6871/15567 (44.1%)	**RR 0.80** (0.78 to 0.81)	555 per 1.000	**111 fewer per 1.000** (from 122 fewer to 105 fewer)

CI, confidence interval; RR, risk ratio.

Explanations

a. Definition of background thyroid status is unclear and, therefore, it is unclear the real prevalence of SCH in both groups.

b. The study aims to examine the effect of levothyroxine in patients with CHF.

c. Cumulative exposure to levothyroxine among treated patients, and background and final TSH values in both groups are unknown.

d. The result could not be clinically relevant.

The bolded text in this table emphasizes the results.

## Discussion

The role of SCH in the pathophysiology of chronic disorders, such as CHF has been well-recognized. Nevertheless, it is unclear whether levothyroxine treatment may have positive or rather detrimental effects on cardiovascular prognosis in this setting.

Current evidence suggests that subclinical thyroid dysfunction, mostly SCH, increases along with advancing age, and the same is for CHF. A relevant proportion of patients diagnosed with both conditions is, hence, expected, especially among elderly patients (>70 years). Timely recognition of thyroid disorders in CHF patients is suggested in order to provide adequate treatments. However, the management of thyroid disorders, including SCH, is based on general recommendations instead of specific findings.

Besides the mechanistic effects of thyroid hormones on myocardial remodeling, cardiac pump efficiency, and hemodynamic (41), the efficacy and effectiveness of lifelong levothyroxine replacement have not been well examined, especially in terms of hard end-points, including CV mortality and CV events. The lack of information is attributable to several aspects that should be considered. First of all, several factors may increase TSH circulating levels, including advanced age (42). This means that not all the cases of TSH elevation should be considered pathological results in most of those diagnosed with SCH and CHF, and a more appropriate cut-off point may be required for defining SCH in the general population and, specifically, in those with CHF. Second, it is unclear what is the ideal TSH value (if one exists) in order to provide beneficial effects and avoid adverse events upon levothyroxine treatment is established in SCH patients with CHF. The evidence suggests that euthyroidism compared to both overt hypothyroidism and SCH in a population with CHF may be protective with respect to adverse CV events, but individualized therapeutic objectives are probably needed considering the large heterogeneity of this population. Third, it is unclear what are the major end-points for defining levothyroxine replacement as appropriate or not. Current evidence, especially in the elderly, suggests starting levothyroxine replacement in case of symptomatic hypothyroidism with the specific aim to attenuate clinical signs and related symptoms and restore baseline quality of life. This suggests that surrogate end-points, indicating improvements in quality of life, and not individualized TSH cut-off points are now the instruments for managing SCH in patients with CHF. Levothyroxine replacement has been demonstrated to improve some surrogate end-points, but this phenomenon does not necessarily reflect improvement in life expectancy and prevention from “hard” CV end-points. Lastly, the benefit and harms attributable to levothyroxine treatment remain unclear, as well as how long it should be the treatment to actually provide beneficial effects.

CHF is also characterized by reduced T3 circulating levels, and the severity of left ventricle dysfunction (i.e., CHF with reduced ejection fraction) is paralleled with the seriousness of T3 deficiency. This condition is also known as the euthyroid sick syndrome, in which existing thyroid hormone imbalance does not reflect on actual hypothalamic-pituitary-thyroid axis dysfunction. The euthyroid sick syndrome is usually observed in acute and chronic severe diseases other than CHF, such as sepsis, undernourishment, and advanced malignancies. The role of the euthyroid sick syndrome in such severe clinical contexts is still debated, as it is unclear whether low T3 levels represent a consequence of the underlying diseases or it may contribute to poor prognosis. At the cardiovascular site, thyroid hormones are essential to sustain cardiocirculatory efficiency. As cardiac myocytes lack substantial levels of deiodinase, intramyocardial conversion of T4 in T3 is negligible; thus, systemic deficiency of T3 substantially reflects on intramyocardial T3 insufficiency.

Observational prospective data suggest that hospitalized patients with heart failure and low T3 levels are at higher risk of all-cause and cardiac mortality than patients with normal levels of T3, thus confirming the protective role of T3 at the myocardial site (43). Although there is no relevant difference in left ventricle ejection fraction, patients with low serum T3 concentration displayed abnormal hemodynamic patterns, such as reduced cardiorespiratory fitness and ventilatory efficiency, and higher levels of natriuretic peptide (43), that are well-recognized factors associated to poor outcomes in patients with CHF (44). In addition, a preclinical study found that exogenous T3 stimulates cardiomyocyte proliferation in murine neonatal cardiomyocytes, suggesting a potential role of T3 supplementation in the myocardial regeneration of injured (adult) heart (45). Based on this assumption, T3 replacement in patients with heart failure and low circulating T3, regardless of any thyroid dysfunction, could have a therapeutic role in improving myocardial efficiency and clinical outcomes. Nevertheless, chronic oral T3 supplementation in CHF patients with mildly reduced ejection fraction and low circulating levels of T3 fails to improve cardiovascular outcomes, and it is not routinely recommended (46). To explain disappointing results, it should be considered that possible discrepancy between circulating and intramyocardial T3 levels exists. This point leads to two specific criticisms. First, as suggested by animal studies, low intramyocardial T3 concentrations were also found when serum T3 levels were in the normal range. Second, serum T3 concentration could not be the best biomarker of tissue T3 concentration, and other surrogate markers should be used for diagnostic purposes and long-term therapeutic monitoring when recurring T3 supplementation. Recently published data in rats indicated an inverse relationship between myocardial and serum T3 concentration and the atrial natriuretic peptide. These data suggest that the reduction of circuiting natriuretic peptide during T3 supplementation in CHF could be a more reliable biomarker of intramyocardial T3 concentration, and it could be used to manage T3 supplementation better and provide more accurate therapeutic goals in CHF (47).

Our systematic review has been conceived to respond to the above-mentioned questions and search focalized on the effects of levothyroxine replacement in a population with CHF and SCH in terms of hard end-points, such as all-cause and CV mortality, CV events, and progression of heart failure. The results showed that most of the evidence in the field is provided by spontaneous trials focusing on surrogate end-points (clinical, laboratory, and echocardiographic paraments) rather than consistent ones.

Two studies were finally included in this systematic review, despite some limitations: one RCT and one observational nationwide cohort study. The two studies are clinically diverse due to different designs, very different study populations, and comparisons, so a meta-analysis appeared inappropriate to provide a reliable synthesis.

In Zijlstra et al., the study population had baseline SCH (TSH 4.6 – 19.9 mUI/mL), but only a minority of them had background CVD (35.9%), and only 38 patients had a history of CHF. Although the objective of this study was to determine the effects of levothyroxine compared to placebo on cardiovascular outcomes in older adults with SCH, the trial was designed to combine data from other two previously published RCTs with different aims (48, 49). In fact, both trials have been designed to assess the efficacy of levothyroxine replacement on quality of life and hypothyroidism-related symptoms in a population of elderly patients. It should be noted that studies assessing cardiovascular safety/efficacy of medication must have a specific design, namely Cardiovascular Outcome Trials (CVOTs). CVOTs are trials specifically designed to assess the impact of medications compared to others or to placebo on the incidence of CV events occurring through the follow-up. CVOTs are normally event-driven studies, and adequate statistical power is achieved after the pre-specified number of CV events occurs. The study duration of CVOTs is, therefore, influenced by baseline CV characteristics of the study population as the number of CV events to achieve in both groups (e.g., treatment versus control) increases alongside the background CV risk. Based on the large experience of CVOTs in other fields of Medicine (i.e., Diabetology), CVOT duration is normally longer than 2-3 years since a lower median follow-up is too short to appreciate an adequate number of CV events. In Zijlstra et al., the number of CV events was 44 (5.4% of the baseline study population) during a median observation of 17 months, and no difference was found between the two groups. Wide interval confidence for each end-point was observed irrespective of the RR value, thus highlighting the existence of great variability in the real effect of levothyroxine compared to placebo (benefit rather than harm). Despite the incidence of CV events appearing similar to epidemiological data in elderly patients (50), the number of CV events was considerably underpowered due to the short follow-up and the restricted number of participants (842).

In Einfeldt et al., data were extracted from five registries (Danish National Patient Register; Civil Registration system records deaths for all Danish citizens; Danish Register of Causes of Death; Danish Register of Medicinal Product Statistics; Danish registers on personal income and transfer payments from the Danish Labor Market) and included participants who had received a diagnosis of heart failure after the first hospital admission. The study purpose was to assess the effect of levothyroxine compared to no treatment on several hard CV end-points, but no information about the background thyroid function was available and, therefore, the number of patients with SCH is unknown. As another important issue, levothyroxine prescription may not be necessary a surrogate of hypothyroidism since levothyroxine could be prescribed to achieve TSH suppression (e.g., differentiated thyroid cancer, thyroid nodules). These data are lacking, affecting possible conclusions (indirectness of results). The statistical relevance of findings in a relative reduction in the risk of all-cause and CV mortality, MACE, and myocardial infarction could be mainly attributable to the considerable sample size of the study population. However, the clinical relevance of these results appears limited, especially considering the scant difference in the absolute risk reduction of observed events and given the increased incidence rate ratios of these events in the group of patients assuming levothyroxine compared with no treatment. Although the two study designs appear as close to the clinical question expressed in our PICO, the results of this systematic review suggest the existence of important limitations in terms of study population (both), intervention (especially Einfeldt et al.), and imprecision of results (especially Zijlstra et al.). Indirectness and imprecision of results characterize the findings of this systematic review, and the overall quality of evidence assessed according to the GRADE methodology is very low.

In conclusion, current data do not draw reliable assumptions in this field, and the lack of evidence did not lead to either recommend/suggest or against the treatment of SCH in CHF patients.

## Conclusion

Specific clinical trials, both randomized and non-randomized intervention trials, are needed to define the role of levothyroxine treatment in patients with SCH and CHF. The study design should include a CVOT with a precise definition of the study population, criteria for both SCH and CHF, and outcomes (e.g., a 3-point major adverse cardiovascular events and frequency of hospital admission due to heart failure). Also, dose-response trials could be useful to potentially identify a better TSH target for a safer prescription of levothyroxine in this clinical setting.

## Author’s note

Scopus id: Vincenzo Triggiani, 6603380071. Antonio Cittadini, 57200895552. Guiseppe Lisco, 57203761410.

## Data availability statement

The raw data supporting the conclusions of this article will be made available by the authors, on reasonable request.

## Author contributions

VT conceived the systematic review; GL and VT searched databases and selected clinical trials after pre-specified critical appraisal; GL drafted the manuscript; VT and AC read the full text and provided feedback. All authors contributed to the article and approved the submitted version.

## Conflict of interest

The authors declare that the research was conducted in the absence of any commercial or financial relationships that could be construed as a potential conflict of interest.

## Publisher’s note

All claims expressed in this article are solely those of the authors and do not necessarily represent those of their affiliated organizations, or those of the publisher, the editors and the reviewers. Any product that may be evaluated in this article, or claim that may be made by its manufacturer, is not guaranteed or endorsed by the publisher.

## Abbreviations

CVDs, CardioVascular Diseases; CVOTs, CardioVascular Outcome Trials; CHF, Chronic Heart Failure; fT4free, free Thyroxine; fT3, Triiodothyronine; GRADE, Grading of Recommendations Assessment Development and Evaluation; MACEOvert, Major Adverse Cardiovascular Events; OH, Hypothyroidism; RTCs, Randomized Clinical Trials; SCH, Subclinical Hypothyroidism; TSH, Thyrotropin Hormone.
