# Supplementary material for: Effect of levothyroxine replacement therapy in patients with subclinical hypothyroidism and chronic heart failure: A systematic review
Source: Front Endocrinol (Lausanne). 2022 Nov 15;13:1013641. doi: 10.3389/fendo.2022.1013641 (PMC9706201; doi:10.3389/fendo.2022.1013641)
Supplement: Supplementary file 1 [file DataSheet_1.pdf]

Supplemental materials

Included studies (2):

1. Einfeldt MN, Olsen AS, Kristensen SL, Khalid U, Faber J, Torp-Pedersen C, Gislason GH, Selmer C. Long-Term Outcome in Patients With Heart Failure Treated With Levothyroxine: An Observational Nationwide Cohort Study. J Clin Endocrinol Metab. 2019 May 1;104(5):1725-1734. doi: 10.1210/jc.2018-01604. PMID: 30517746.
2. Zijlstra LE, Jukema JW, Westendorp RGJ, Du Puy RS, Poortvliet RKE, Kearney PM, O'Keeffe L, Dekkers OM, Blum MR, Rodondi N, Collet TH, Quinn TJ, Sattar N, Stott DJ, Trompet S, den Elzen WPJ, Gussekloo J, Mooijaart SP. Levothyroxine Treatment and Cardiovascular Outcomes in Older People With Subclinical Hypothyroidism: Pooled Individual Results of Two Randomised Controlled Trials. Front Endocrinol (Lausanne). 2021 May 20;12:674841. doi: 10.3389/fendo.2021.674841. PMID: 34093444; PMCID: PMC8173189.

Study number 2 is a pooled analysis from two RCTs:

A. Stott DJ, Rodondi N, Kearney PM, Ford I, Westendorp RGJ, Mooijaart SP, Sattar N, Aubert CE, Aujesky D, Bauer DC, Baumgartner C, Blum MR, Browne JP, Byrne S, Collet TH, Dekkers OM, den Elzen WPJ, Du Puy RS, Ellis G, Feller M, Floriani C, Hendry K, Hurley C, Jukema JW, Kean S, Kelly M, Krebs D, Langhorne P, McCarthy G, McCarthy V, McConnachie A, McDade M, Messow M, O'Flynn A, O'Riordan D, Poortvliet RKE, Quinn TJ, Russell A, Sinnott C, Smit JWA, Van Dorland HA, Walsh KA, Walsh EK, Watt T, Wilson R, Gussekloo J; TRUST Study Group. Thyroid Hormone Therapy for Older Adults with Subclinical Hypothyroidism. N Engl J Med. 2017 Jun 29;376(26):2534-2544. doi: 10.1056/NEJMoa1603825. Epub 2017 Apr 3. PMID: 28402245.

B. Mooijaart SP, Du Puy RS, Stott DJ, Kearney PM, Rodondi N, Westendorp RGJ, den Elzen WPJ, Postmus I, Poortvliet RKE, van Heemst D, van Munster BC, Peeters RP, Ford I, Kean S, Messow CM, Blum MR, Collet TH, Watt T, Dekkers OM, Jukema JW, Smit JWA, Langhorne P, Gussekloo J. Association Between Levothyroxine Treatment and Thyroid-Related Symptoms Among Adults Aged 80 Years and Older With Subclinical Hypothyroidism. JAMA. 2019 Nov 26;322(20):1977-1986. doi: 10.1001/jama.2019.17274. PMID: 31664429; PMCID: PMC6822162.

Language complaints and missing information (1, no abstract and full text publicly available and no additional data have been collected after contacting the authors on April 17, 2022):

1. Ageev FT, Blankova ZN, Seredenina EM, Riabtseva OIu, Ovchinnikov AG, Svirida ON. [Efficacy and safety of hormone replacement therapy with levothyroxine in patients with subclinical hypothyroidism and heart failure]. Kardiologiia. 2011;51(5):70-4. Russian. PMID: 21649597.

Study protocols (6):

1. Du Puy RS, Postmus I, Stott DJ, Blum MR, Poortvliet RKE, Den Elzen WPJ, Peeters RP, van Munster BC, Wolffenbuttel BHR, Westendorp RGJ, Kearney PM, Ford I, Kean S, Messow CM, Watt T, Jukema JW, Dekkers OM, Smit JWA, Rodondi N, Gussekloo J, Mooijaart SP. Study protocol: a randomised controlled trial on the clinical effects of levothyroxine treatment for subclinical hypothyroidism in people aged 80 years and over. BMC Endocr Disord. 2018 Sep 19;18(1):67. doi: 10.1186/s12902-018-0285-8. PMID: 30231866; PMCID: PMC6146605.

2. Stott DJ, Gussekloo J, Kearney PM, Rodondi N, Westendorp RG, Mooijaart S, Kean S, Quinn TJ, Sattar N, Hendry K, Du Puy R, Den Elzen WP, Poortvliet RK, Smit JW, Jukema JW, Dekkers OM, Blum M, Collet TH, McCarthy V, Hurley C, Byrne S, Browne J, Watt T, Bauer D, Ford I. Study protocol; Thyroid hormone Replacement for Untreated older adults with Subclinical hypothyroidism - a randomised placebo controlled Trial (TRUST). BMC Endocr Disord. 2017 Feb 3;17(1):6. doi: 10.1186/s12902-017-0156-8. PMID: 28158982; PMCID: PMC5291970.
3. Zhang X, Wang WY, Zhang K, Tian J, Zheng JL, Chen J, An SM, Wang SY, Liu YP, Zhao Y, Wang JJ, Yang M, Tang YD. Efficacy and safety of levothyroxine (L-T4) replacement on the exercise capability in chronic systolic heart failure patients with subclinical hypothyroidism: Study protocol for a multi-center, open label, randomized, parallel group trial (ThyroHeart-CHF). Trials. 2019 Feb 19;20(1):143. doi: 10.1186/s13063-019-3219-5. PMID: 30782213; PMCID: PMC6381733.
4. Single centre randomized study to evaluate the effect of levothyroxine on cardiac function in patients with chronic heart failure and subclinical hypothyroidism – LEVOTHYROXINE.
5. IEMO 80-plus thyroid trial EUCTR2012-004160-22-NL.
6. IEMO 80-plus thyroid trial NTR3851.
